# S‐100B as an extra selection tool for FDG PET/CT scanning in follow‐up of AJCC stage III melanoma patients

**DOI:** 10.1002/jso.25682

**Published:** 2019-08-29

**Authors:** Eric A. Deckers, Kevin P. Wevers, Anneke C. Muller Kobold, Samantha Damude, Otis M. Vrielink, Robert J. van Ginkel, Lukas B. Been, Barbara L. van Leeuwen, Harald J. Hoekstra, Schelto Kruijff

**Affiliations:** ^1^ Department of Surgical Oncology, University Medical Center Groningen University of Groningen Groningen The Netherlands; ^2^ Department of Laboratory Medicine, University Medical Center Groningen University of Groningen Groningen The Netherlands

**Keywords:** biomarker, FDG PET/CT, follow‐up, melanoma, recurrence, S‐100B

## Abstract

**Background and Objectives:**

This current study assessed the value of S‐100B measurement to guide fluorodeoxyglucose (FDG) positron emission tomography/computed tomography (PET/CT) scanning for detecting recurrent disease in stage III melanoma patients.

**Methods:**

This study included 100 stage III melanoma patients in follow‐up after curative lymph node dissection. Follow‐up visits included physical examination and S‐100B monitoring. FDG PET/CT scanning was indicated by clinical symptoms and/or elevated S‐100B.

**Results:**

Of 100 patients, 13 (13%) had elevated S‐100B without clinical symptoms, of whom 7 (54%) showed disease evidence upon FDG PET/CT scanning. Twenty‐six patients (26%) had clinical symptoms with normal S‐100B and FDG PET/CT revealed metastasis in 20 (77%). Three patients had clinical symptoms and elevated S‐100B, and FDG PET/CT revealed metastasis in all three (100%). Overall, FDG PET/CT scanning revealed metastasis in 30 of the 42 patients (71.4%). For seven recurrences, elevated S‐100B prompted early detection of asymptomatic disease; 10% of all asymptomatic patients in follow‐up, 23% of all patients with recurrent disease.

**Conclusion:**

S‐100B cannot exclude recurrent disease during follow‐up of stage III melanoma. However, adding S‐100B measurement to standard clinical assessment can guide FDG PET/CT scanning for detecting recurrent melanoma.

## 1 INTRODUCTION

The incidence of cutaneous melanoma has increased worldwide over recent decades.^1^ In the Netherlands, 1563 new cases were diagnosed in 1990, and this number grew to 6743 in 2017.^2^ Mortality has increased at a lower rate, with 348 melanoma‐related deaths in 1990 in the Netherlands, and 767 in 2016. The lower rise in mortality is because the increased incidence largely involves more cases of thin melanoma, likely due to improved awareness and earlier melanoma detection.^1^, ^3^

In melanoma patients, the goal of follow‐up surveillance is the cost‐effective detection of recurrence at an early stage, based on the assumption that early surgical and/or systemic treatment will improve disease‐free survival (DFS), melanoma‐specific survival (MSS), and overall survival (OS). There are no clinical data to support this assumption. Until now, data on the effectiveness of routine imaging for recurrence detection in follow‐up is limited. Data with respect to an impact on the quality of life in melanoma patients with intensive follow‐up schedules are lacking.^4^

The melanoma biomarker S‐100B reportedly shows strong correlations with distant metastasis‐free survival and OS in stage IIB‐III melanoma patients.^5^ The serum concentration of S‐100B is correlated with disease stage, and S‐100B is an independent predictor of melanoma prognosis in patients undergoing therapeutic lymph node dissection (TLND) for nodal macro‐metastases.^6^, ^7^ German melanoma follow‐up guidelines added the melanoma biomarker S‐100B and Italian guidelines added both S‐100B and fluorodeoxyglucose (FDG) positron emission tomography/computed tomography (PET/CT) scanning, in addition to regular patient history and physical examination.^8^, ^9^ Specifically, S‐100B measurement has been recommended for use in some follow‐up guidelines in the selection of stage III patients to undergo FDG PET/CT scanning. However, the added value of this screening is unknown.^10^, ^11^ Assessment of the melanoma marker could potentially contribute to the detection of asymptomatic disease recurrence in stage III melanoma, and therewith reduce the number of routine FDG PET/CT scans. As long as scientific data on the effect of standard scanning regimens are lacking, a strategy using a biomarker as a trigger for scanning in asymptomatic patients could be an interesting alternative.

In the present study, we primarily aimed to assess the added value of the biomarker S‐100B as a selection tool before FDG PET/CT scanning for the detection of recurrent disease in stage III melanoma patients. Our secondary objective was to evaluate the associated costs of this follow‐up strategy.

## 2 MATERIALS AND METHODS

### 2.1 Patients

This investigation included all patients with stage III melanoma who underwent curative treatment with complete lymph node dissection (CLND) for a positive sentinel node, or with TLND for macro‐metastases, and were treated at the Division of Surgical Oncology of the University Medical Center Groningen (UMCG), the Netherlands. The study protocol was applied to all stage III melanoma patients who were in follow‐up in 2015, and to all newly diagnosed patients since 2015. Study data were collected during the period 2015‐2018. Patients who underwent off‐protocol FDG PET/CT imaging during this time period were excluded from the present analysis. Data collection was conducted according to the declaration of Helsinki ethical principles for medical research involving human subjects.^12^

### 2.2 Follow‐up

Outpatient follow‐up visits included patient medical history, physical examination, and serum S‐100B and LDH laboratory testing following the UMCG protocol (Table 1).

**Table 1:** Follow‐up protocol for stage III melanoma at UMCG

Years of follow‐up	Outpatient visit + S‐100B measurement
1st year	4× per year
2nd year	3× per year
3rd‐5th year	2× per year
>5th year	1× per year

Abbreviation: UMCG, University Medical Center Groningen.

Serum S‐100B level laboratory calculations were performed as previously described.^7^ The S‐100B cut‐off value was ≥0.15 µg/L. S‐100B level was defined as borderline if it was between 0.10 and 0.15 µg/L and/or showed a ≥40% elevation compared to the last measurement. A change of ≥40% was considered statistically significant based on the biological and analytical variations of S‐100B.^13^

**Figure 1:**
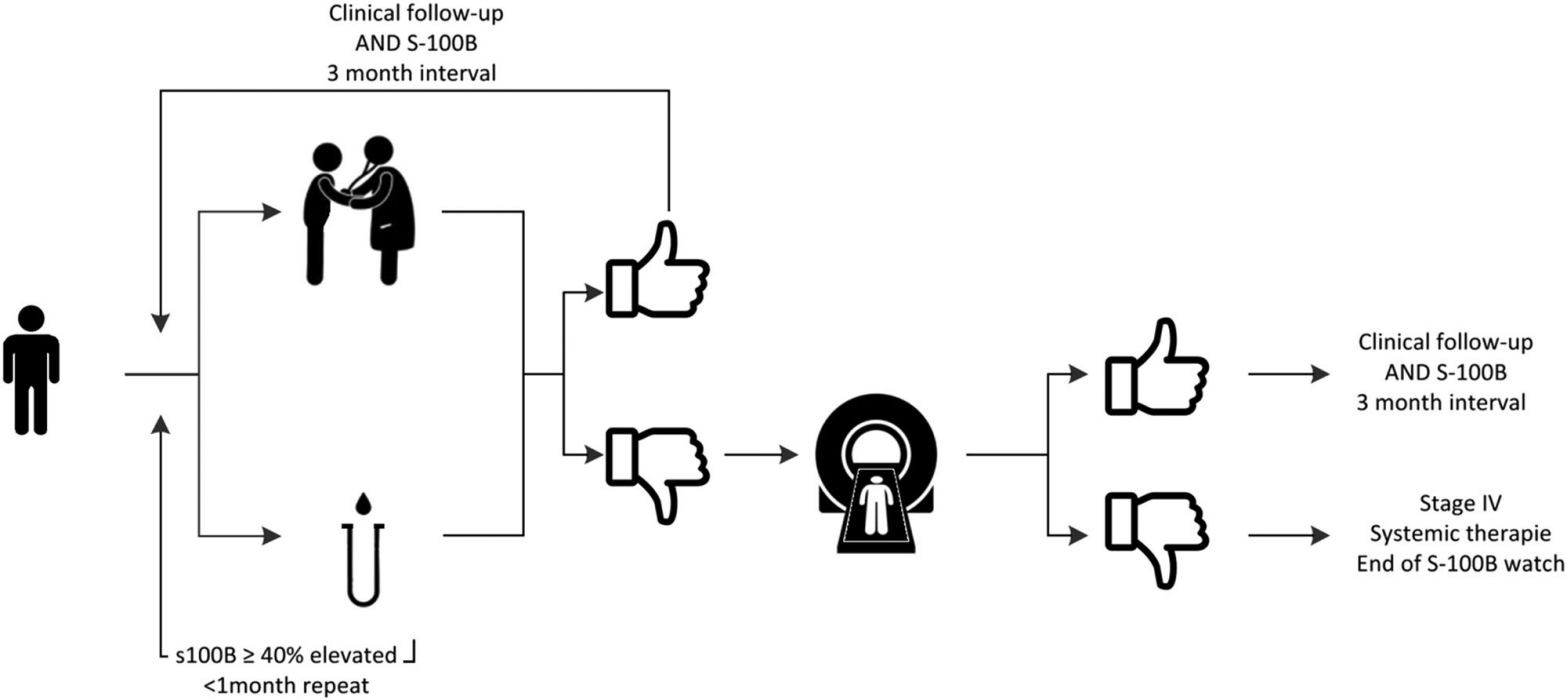
Clinical follow‐up and S‐100B measurement, 3‐month interval

FDG PET/CT scanning was performed in cases with clinical suspicion of recurrent melanoma and/or an elevated S‐100B level. In cases with borderline S‐100B values, measurement was repeated after 4 weeks, and FDG PET/CT scanning was performed when S‐100B was persistently borderline or elevated (Figure 1). The indication for FDG PET/CT scanning was recorded, and categorized into three groups: (a) clinical symptoms and normal S‐100B, (b) clinical symptoms and elevated S‐100B, and (c) no clinical symptoms and elevated S‐100B.

### 2.3 Costs

For all patients participating in the UMCG follow‐up protocol, we calculated the follow‐up costs of the detection of asymptomatic and symptomatic recurrences, including S‐100B measurement, as well as the total costs of FDG PET/CT scanning. Data were acquired from the Patient Financial Department of the UMCG.

## 3 RESULTS

### 3.1 Patients

A total of 122 patients with stage III melanoma were in follow‐up during the study period. The median follow‐up after CLND or TLND was 4.7 years (0.7‐15.3 years). We excluded 22 patients due to off‐protocol FDG PET/CT scanning. Of the remaining 100 patients, 52 were male and 48 were female, and the median age was 57 years (range, 25‐89 years) (Table 2). During the study period, the 100 patients attended a total of 456 outpatient visits with corresponding S‐100B measurements (Table 3).

**Table 2:** Baseline characteristics of patients in the follow‐up cohort

Characteristic	
Sex	
Female, n, %	48 (48.0%)
Male, n, %	52 (52.0%)
Years of age, median (range)	57 (25‐89)
Primary melanoma site, n, %	
Head	4 (4%)
Trunk/back	36 (36%)
Lower extremity	41 (41%)
Upper extremity	15 (15%)
Unknown primary	4 (4%)
Breslow thickness in mm, median (range)	2.0 (0.4‐14.0)
Ulceration	
Yes	32 (32%)
No	52 (52%)
Sentinel node performed	
Yes	69 (69%)
No	26 (26%)
Sentinel node positive	
Yes	65 (94%)
No	4 (6%)
Lymph node dissection	
CLND	43 (43%)
TLND	36 (36%)
Type of melanoma, n, %	
Superficial spreading	64 (64%)
Nodular melanoma	21 (21%)
Verrucous nevoid melanoma	1 (1%)
Spitzoid melanoma	1 (1%)
Other	13 (13%)

Abbreviations: CLND, completion lymph node dissection; TLND, therapeutic lymph node dissection.

**Table 3:** Overview of follow‐up visits, S‐100B tests, and FDG PET/CT scans

Patient assessment	
Years of follow‐up, median (range)	4.7 (0.7‐15.3)
S‐100B samples, N	456
Normal, n, %	414 (90.8%)
Elevated,^a^ n, %	42 (9.2%)
Indication for FDG PET/CT scan, n, %	
Symptoms	26 (62%)
Symptoms + elevated S‐100B	3 (7.1%)
Elevated S‐100B	10 (23.8%)
S‐100B level elevation ≥40%	3 (7.1%)
Total FDG PET/CT scans,^b^ N	42
Positive FDG PET/CT scans, n (%)	30 (71.4%)
Negative FDG PET/CT scans, n (%)	12 (28.6%)

Abbreviations: FDG, fluorodeoxyglucose; PET/CT, positron emission tomography/computed tomography.

^a^ All elevated S‐100B samples, including repeated measurements from a single patient in cases showing an S‐100B elevation of ≥40%.

^b^ One FDG PET/CT scan per patient; additional scans performed after one positive FDG PET/CT scan were not counted.

### 3.2 Indications for PET/CT

During the 456 outpatient visits, elevated S‐100B was found 42 times (9.2%) (Table 3). Of the 100 patients, 58 patients (58%) had no clinical suspicion of recurrence or elevated S‐100B level during their follow‐up visits, and thus had no indication for FDG PET/CT scanning. The remaining 42 patients (42%) had clinical symptoms and/or elevated S‐100B and, therefore an indication for FDG PET/CT scanning. Thirteen patients were asymptomatic but had elevated S‐100B levels (in 54% recurrent melanoma on PET/CT). Twenty‐six patients presented with clinical symptoms and a normal S‐100B level (in 77% recurrence on PET/CT). Three patients had both clinical symptoms and elevated S‐100B (100% recurrence on PET/CT) (Table 4).

**Table 4:** Indications for FDG PET/CT scanning and their association with recurrent disease

	Positive FDG PET/CT scan (n = 30)		Negative FDG PET/CT scan (n = 12)	
Indication for FDG PET/CT scan		Symptomatic vs asymptomatic		Symptomatic vs asymptomatic
Symptoms (n = 26)	20 (76.9%)	23 (77%)	6 (23.1%)	6 (50%)
Symptoms + S‐100B (n = 3)	3 (100%)		0 (0%)	
Elevated S‐100B (n = 13)	7 (53.8%)	7 (23%)	6 (46.2%)	6 (50%)

Abbreviations: FDG, fluorodeoxyglucose; PET/CT, positron emission tomography/computed tomography.

Of all 100 patients, 26 had symptoms without S‐100B elevation, which leaves 74 asymptomatic patients in this cohort. Thirteen of these asymptomatic patients (18%) had elevated S‐100B levels and seven (10%) showed recurrent disease on the FDG PET/CT scan.

### 3.3 Yield per PET/CT indication

**Figure 2:**
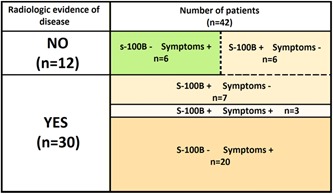
PET outcome proportionally classified for indication for 42 of 100 scanned patients. PET, positron emission tomography [Color figure can be viewed at wileyonlinelibrary.com]

A total of 42 FDG PET/CT scans were obtained in this study, of which 30 (71%) showed evidence of recurrent disease. Of these 30 disease‐revealing FDG PET/CT scans, 7 (23%) were performed based on elevated S‐100B levels in asymptomatic patients. The remaining 23 disease‐revealing scans were performed based on clinical symptoms (77%), 3 with and 20 without elevated S‐100B measurements. Twelve FDG PET/CT scans were negative, 6/29 symptomatic patients (21%) (with and without elevated S‐100B) and 6/13 patients with elevated S‐100B (46%) (*P = *.09) (Table 4, Figure 2).

### 3.4 Stage and recurrence pattern

Of the 30 disease‐revealing FDG PET/CT scans, 15 patients were initially diagnosed with (AJCC version 8) stage IIIA disease, 8 with stage IIIB, 5 with stage IIIC and 2 with stage IIID. The 12 negative FDG PET/CT scans included 2 stage IIIA, 7 stage IIIB and 3 stage IIIC patients.

Differences in recurrence pattern were found for the 20 symptomatic and the 7 asymptomatic patients. Of the 20 symptomatic patients, 12 (60%) presented with locoregional recurrences, 5 (25%) with distant recurrences, and 3 (15%) with both locoregional and distant recurrences. For asymptomatic patients scanned for high S‐100B, five of seven patients (71.4%) had distant and two patients (28.6%) locoregional metastases.

### 3.5 Costs

The total S‐100B laboratory costs and the costs of FDG PET/CT scanning for all 100 stage III melanoma patients undergoing follow‐up under the UMCG protocol were calculated. In 2015, the cost of processing a single S‐100B sample was €109 and the cost of a FDG PET/CT scan was €913. The total cost was €88.050 for all S‐100B samples (456 in total) processed during follow‐up of 100 patients plus the cost of the 42 FDG PET/CT scans.

When a standard scan protocol (eg, as suggested in the TRIM [A Randomized Trial to Assess the Role of Imaging During Follow up After Radical Surgery of High Risk Melanoma] study [NCT03116412]) is applied to the same cohort with corresponding follow‐up and costs as in the current study, total diagnostic costs (FDG PET/CT and S‐100B) would have been €408.800 (100 patients in follow‐up with S‐100B and FDG PET/CT at baseline, 86 patients at 6 months, 78 patients at 12 months, 69 patients at 24 months and 67 patients at 36 months).

## 4 DISCUSSION

The present study evaluated the tumor marker S‐100B in stage III melanoma patients as an additional tool to guide FDG PET/CT scanning for the detection of recurrent disease. Of all S‐100B measurements, 2.9% eventually led to FDG PET/CT scanning. However, S‐100B was the only trigger for the FDG PET/CT scan in 23% of all patients in whom recurrent disease was detected. For all asymptomatic patients in follow‐up, S‐100B measurement led to the discovery of recurrent disease in 10% of them. Clearly, S‐100B measurement cannot exclude disease during follow‐up of stage III melanoma. However, our findings show that the tumor marker can serve as an extra tool, in addition to standard clinical assessment, to guide FDG PET/CT scanning for the detection of recurrent disease, without the financial, logistical, and radiation burdens of a standard scanning follow‐up scheme.

In cases of cutaneous melanoma, S‐100B serum concentrations are a prognostic marker of metastatic disease.^5^, ^7^ Serum concentrations of S‐100B correlate with disease stage, although large variation is observed with or without S‐100B elevation.^6^ Previous findings suggest that S‐100B levels may be influenced by the melanoma metastasis location and by variations in the ability of melanoma cells to produce S‐100B.^14^, ^15^, ^16^ Together with the limited S‐100B elevation in patients with low tumor load, it is difficult to designate S‐100B as a solid indicator of recurrence.^17^

In the current study, disease recurrence was detected on FDG PET/CT scans that were performed in 7 patients (23%) with elevated S‐100B and no clinical symptoms, in 3 patients (10%) with clinical symptoms and elevated S‐100B, and in 20 patients (67%) with clinical symptoms and normal S‐100B. These data correspond with previous findings that elevated S‐100B was the only sign in 20% of patients with disease progression.^16^ In the present series, 33% of patients that recurred IV disease had increased S‐100B, which is in line with prior reports of increased S‐100B levels in 4% to 100% of patients with stage IV disease.^6^ In stage II and III melanoma patients, the reported sensitivity and specificity of S‐100B for recurrent disease varies from 29% to 43% and 93% to 94%, respectively.^7^, ^11^, ^18^

To compare with other tumor markers, the widely accepted colorectal cancer biomarker carcino‐embryonic antigen (CEA) has a 41% to 97% sensitivity, which is somewhat higher, and a 52% to 100% specificity, which is comparable to that of S‐100B.^19^ A recent study revealed that 1.5% of all CEA measurements from curatively treated patients with stage I‐III colorectal cancer ultimately led to recurrence detection.^20^, ^21^ As with S‐100B, a normal CEA level does not exclude recurrent disease.^22^

Tumor markers can be used in cancer detection and diagnosis, but are mainly used in follow‐up to detect recurrent disease in an early phase.^23^ The recent development of successful systemic treatment options for stage IV melanoma have given rise to a greater need for early detection of recurrence. It remains unclear whether earlier diagnosis and treatment of stage IV disease with immune or targeted therapy further contributes to improved MSS and OS rates, as lead‐time bias may occur.^24^, ^25^ Recent literature suggests a routine substage‐III‐specific FDG PET/CT schedule for asymptomatic detection of recurrences. However, the same lead‐time bias argument as for biomarkers might be applicable.^26^ A randomized trial is required to determine whether the gained time reflects real survival time or just earlier knowledge of disease. At the present time, it is clear that adjuvant therapy has advantages over therapies in metastatic settings, and that more durable responses and improved long‐term survival are observed with low tumor load.^27^, ^28^, ^29^, ^30^

The Swedish Melanoma Study Group has initiated a trial investigating the effectiveness of standard imaging in Sweden (TRIM study; NCT03116412). This prospective randomized multicenter study of the roles of imaging and laboratory testing during follow‐up after radical surgery of stage IIB‐III melanoma was proposed in 2017, with OS as the study endpoint. Based on the scheduled outpatient visits, with corresponding FDG PET/CT scans (€913) and S‐100B samples (€109), the follow‐up costs for 100 patients using the TRIM protocol would be €408.800 compared to the cost of €88.050 in our current study. Compared to the UMCG protocol applied in our present study, the standard scanning proposed in the TRIM study might lead to earlier detection of metastases, but would also greatly increase melanoma follow‐up costs and the radiation burden. Moreover, the additional scans would lead to incidental findings not contributing to melanoma treatment or disease‐related survival.^31^ The current study protocol could reduce FDG PET/CT scans in asymptomatic melanoma patients, thereby reducing their radiation exposure and the total follow‐up costs compared to a standard scanning protocol. However, one must be aware that normal S‐100B levels do not exclude metastatic disease, emphasizing the importance of thorough self‐inspection by patients and physical examination during follow‐up visits.

There are guidelines, based on AJCC version 8, that advice stage IIIC and IIID often receive routine scans, sometimes even stage IIIB.^11^, ^32^ Most patients, who have undergone a FDG PET/CT scan in this study were stage IIIA or IIIB. This means using S‐100B in selecting for FDG PET/CT scan results in a more refined follow‐up system.

This study has limitations. First, most patients were included retrospectively and on‐protocol follow‐up was 3 years as the median follow‐up since stage III diagnosis was 4.7 years. This makes the population more heterogeneous and might influence the recurrence risk. It could be one reason for the slightly lower number of recurrences (30%) than the 38% reported in a recent published study that used routine, substage‐specific stage III PET/CT scanning schedule.^26^ Secondly, the present study cannot determine the exact survival gain associated with earlier stage IV diagnosis, or the effect of lead‐time bias. In addition, it is difficult to define what the exact gained S‐100B detection percentage is. When the detection rate is calculated over all followed asymptomatic stage III patients the percentage would be 10% (7/71). However, a biomarker can never detect recurrent disease in those patients that in fact do not have a recurrence. When the gain is calculated for the patients that during this study proved recurr (n = 30), this number is 23% (7/30), which could be an overestimation because there might still have been patients is the study follow‐up with occult recurrent disease.

Therefore, we conclude that the addition of S‐100B measurement in the follow‐up of stage III melanoma prompted detection of stage IV disease in 10% of all asymptomatic stage III patients, and resulted in 23% additional upstaging. Without the use of S‐100B there would have been no indication for FDG PET/CT scanning in this 10% of asymptomatic patients, and 23% of all recurrences would have been found later. In an era with expanding possibilities for systemic melanoma treatment and where routine scanning is a contested practice, there is growing demand for earlier stage IV diagnosis. Adding S‐100B measurement to follow‐up could be a way to support this demand, when patients are still asymptomatic. Future research is needed to optimize its use, to assess the absolute survival gain, and compare to the efficacy and costs of this follow‐up method with those of standard scanning protocols. Research should also focus in the future on patient and tumor characteristics that may predict the sensitivity of S‐100B during follow‐up, with the aim of identifying patient subgroups in which S‐100B shows higher sensitivity, to maximize the effectiveness of this tool.

## 5 CONCLUSIONS

S‐100B cannot exclude recurrent disease during follow‐up of stage III melanoma. However, adding S‐100B measurement to standard clinical assessment can effectively guide FDG PET/CT scanning for detecting recurrent melanoma. Future studies are needed to determine whether this protocol is a good alternative to follow‐up regimens that include standard scheduled FDG PET/CT scans.

## CONFLICT OF INTERESTS

The authors declare that there are no conflict of interests.

## DATA ACCESSIBILTY

The data that support the findings of this study are available on request from the corresponding author. The data are not publicly available due to privacy or ethical restrictions.

## SYNOPSIS

S‐100B cannot exclude recurrent disease in the follow‐up of stage III melanoma, but might be useful as an extra selection tool in FDG PET/CT scanning for the detection of recurrent disease.
